# NRF2 signaling pathway and telomere length in aging and age-related diseases

**DOI:** 10.1007/s11010-023-04878-x

**Published:** 2023-11-02

**Authors:** Alessandro Medoro, Luciano Saso, Giovanni Scapagnini, Sergio Davinelli

**Affiliations:** 1 Department of Medicine and Health Sciences “V. Tiberio”, University of Molise, Via F. De Sanctis, s.n.c., 86100 Campobasso, Italy; 2 Department of Physiology and Pharmacology “Vittorio Erspamer”, Sapienza University of Rome, Rome, Italy

**Keywords:** NRF2, Telomere length, Oxidative stress, Aging

## Abstract

The transcription factor nuclear factor erythroid 2-related factor 2 (NRF2) is well recognized as a critical regulator of redox, metabolic, and protein homeostasis, as well as the regulation of inflammation. An age-associated decline in NRF2 activity may allow oxidative stress to remain unmitigated and affect key features associated with the aging phenotype, including telomere shortening. Telomeres, the protective caps of eukaryotic chromosomes, are highly susceptible to oxidative DNA damage, which can accelerate telomere shortening and, consequently, lead to premature senescence and genomic instability. In this review, we explore how the dysregulation of NRF2, coupled with an increase in oxidative stress, might be a major determinant of telomere shortening and age-related diseases. We discuss the relevance of the connection between NRF2 deficiency in aging and telomere attrition, emphasizing the importance of studying this functional link to enhance our understanding of aging pathologies. Finally, we present a number of compounds that possess the ability to restore NRF2 function, maintain a proper redox balance, and preserve telomere length during aging.

## Introduction

Aging is a biological process that is characterized by a gradual decline in cellular and physiological functions. Many factors contribute to aging, including the accumulation of macromolecular damage in response to a variety of environmental and endogenous stressors. This accumulation of the damage over time and across tissues is a primary risk factor in the genesis of many age-related diseases, such as cardiovascular disease (CVD), cancer, neurodegeneration, diabetes, osteoporosis, and sarcopenia [1, 2]. There is currently evidence that the rate of biological aging is driven by an interconnected network of molecular biological processes that contributes to the development of age-related conditions. However, many of these processes, such as genomic instability, mitochondrial dysfunction, and telomere shortening, are associated with chronic oxidative stress caused by elevated levels of reactive oxygen species (ROS) [3, 4]. Oxidative stress occurs when the cellular balance between the production of ROS and antioxidant defenses is disrupted. This altered redox status is accompanied by deleterious effects on cell survival, including oxidative modifications on biological macromolecules. Although there is a growing consensus that ROS act as mediators in a variety of signaling pathways, during aging, accumulation of defective/damaged organelles, especially mitochondria, leads to uncontrolled levels of ROS production. Likewise, prolonged exposure to xenobiotics or excessive production of pro-inflammatory cytokines may increase the levels of intracellular ROS and contribute to cell injury through oxidative damage [5, 6]. Under conditions of oxidative stress, ROS causes various types of genotoxic stress, including dual damage at telomeres and mitochondria [7].

All aerobic organisms have evolved critical defense mechanisms to combat the harmful effects of intrinsic and extrinsic oxidative insults and preserve homeostatic functions. Although numerous endogenous antioxidant systems are in place to scavenge and maintain proper ROS levels, the activation of nuclear factor erythroid 2-related factor 2 (NFE2L2; more commonly known as NRF2) plays a pivotal role in the maintenance of the cellular redox homeostasis. NRF2 is a basic leucine zipper (bZIP) transcription factor with a cap ‘n’ collar (CNC) structure. It is ubiquitously expressed and present in various organs, including the kidney, muscle, lung, brain, liver, and heart [8]. Under basal conditions, NRF2 is tightly regulated by Kelch-like ECH-associated protein 1 (KEAP1), a repressor protein that mediates the degradation of NRF2 by the ubiquitin–proteasome pathway. Once activated by oxidative stress, NRF2 translocates to the nucleus where it binds to antioxidant response elements (ARE) in the promoter region of genes involved in redox regulation, xenobiotic metabolism, DNA repair, apoptosis, proteostasis, and regulation of inflammation [9]. In addition to its roles in defending cells against electrophilic insults and oxidative damage, NRF2 may be a modulator of longevity-related pathways associated with longer lifespan and healthspan in long-lived species and humans with exceptional longevity [10–12]. However, mounting evidence suggests a gradual reduction of NRF2 with age, leading to unmitigated oxidative stress and contributing to the aging phenotype. Furthermore, an age-associated loss of NRF2 function may play a role on many of the cellular and molecular processes known to drive aging (i.e., hallmarks of aging), including cellular senescence, mitochondrial dysfunction, and telomere attrition [13, 14].

Among the many molecular changes associated with old age, telomere length (TL) has been recognized as one of the best biomarkers of aging. Despite pre-analytical and analytical issues, hampering the implementation of TL as routine marker into clinical practice, human studies have found a significant inverse relationship between TL and several age-related conditions [15, 16]. Telomeres are the genomic portions at the ends of linear chromosomes. They are specialized structures that maintain the structural integrity of chromosomes. However, at each somatic cell division cycle, human telomeres lose 50–200 base pairs due to incomplete synthesis of the lagging strand during the DNA replication [17]. In addition, data from several studies indicate that telomeric DNA is hypersensitive to oxidative damage, a phenomenon recently named TelOxidation [18]. Accordingly, enhanced levels of oxidative stress may cause an accelerated telomere shortening, increasing the rate of developing age-related diseases [19]. It has been proposed that oxidative stress and free radicals play a crucial role in telomere shortening by altering both telomerase activity and telomere repeat binding factor 2 (TRF-2) expression levels [20].

As NRF2 plays a crucial role in protecting cells from oxidative damage, an age-related decline in NRF2 function could be a key driving force behind telomere attrition. Here, we provide an overview of the molecular basis regulating the functional link between NRF2 and telomeres in aging and age-related diseases, also highlighting novel strategies to preserve their role in cellular homeostasis.

## Role of NRF2 in oxidative stress

All eukaryotic organisms have evolved sophisticated mechanisms to manage oxidative and electrophilic insults that arise from internal metabolic reactions or xenobiotic challenges. NRF2 has evolved over millennia to become a versatile transcription factor that induces a broad range of biological responses, including detoxification and antioxidant activities. Recent advances in our understanding of the NRF2 pathway have revealed that this system is related to a number of age-related diseases, such as cancer, neurodegenerative diseases, and diabetes mellitus [21, 22].

**Fig. 1:**
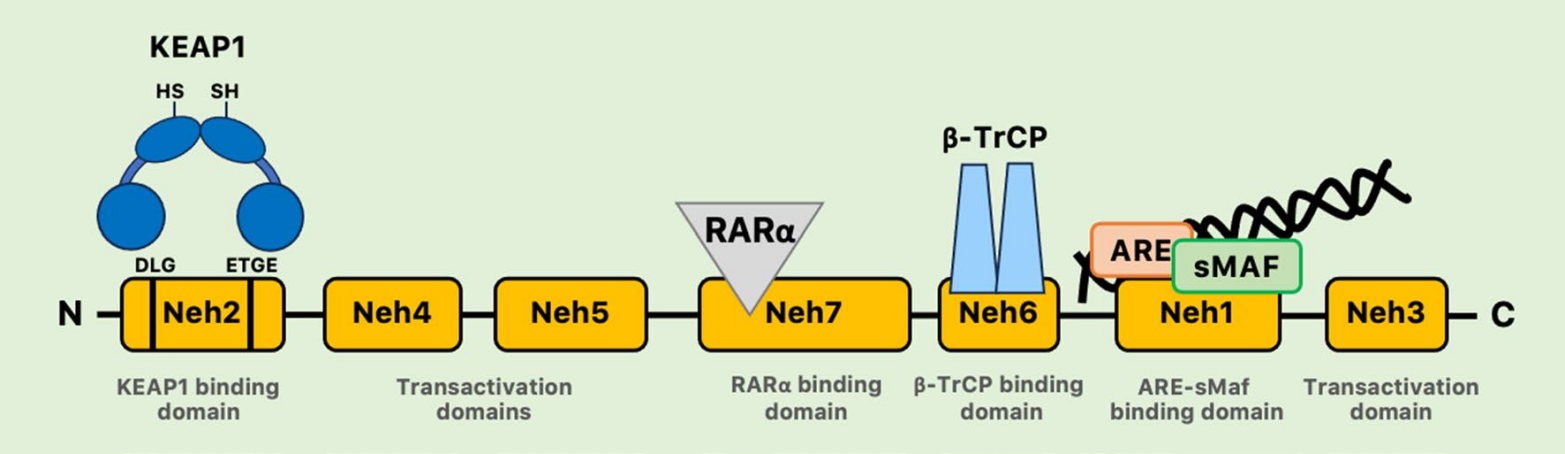
Functional domains of human NRF2. NRF2 has seven highly conserved domains (Neh1–Neh7). Neh1 contains a CNC basic-region leucine zipper domain that interacts with sMAF and DNA. Neh2 contains two recognition motifs (ETGE and DLG), which are required for binding with KEAP1. Neh3, Neh4, and Neh5 domains are necessary for transactivation. Neh6 is involved in a KEAP1-independent degradation of NRF2 by binding β-TrCP. Neh7 interacts with RARα suppressing the activity of NRF2. *ARE* antioxidant response elements, *β-TrCP* binding β-transducin repeat-containing protein, *CNC* cap ‘n’ collar, *KEAP1* kelch-like ECH-associated protein 1, *Neh* NRF2-ECH homology domains, *RARα* retinoic acid receptor α, *sMAF* small MAF proteins

The NRF2 signaling pathway can regulate more than 600 genes and some of these genes encode cytoprotective proteins that are also associated with regulation of inflammatory response [9]. In humans, NRF2 is composed of 605 amino acids and 7 highly conserved NRF2-ECH homology domains (Neh 1–7) (Fig. 1). The Neh1 domain binds to the ARE sequence using a bZIP motif [23]. In this region, NRF2 will heterodimerize with small musculoaponeurotic fibrosarcoma proteins (sMAF) to induce the transcription of NRF2 target genes. The Neh2 domain interacts with Keap1 using the DLG (Asp-Leu-Gly) and ETGE (Glu-Thr-Gly-Glu) motifs, while Neh 3, 4, and 5 domains are required to promote the transactivation of several cytoprotective genes. The Neh6 domain controls the degradation of NRF2 by binding β-transducin repeat-containing protein (β-TrCP) and with a KEAP1-independent mechanism. The Neh7 domain interacts with the retinoic acid receptor α (RARα), a nuclear receptor that is known to suppress the activity of NRF2 pathway and decrease ARE gene expression [24].

Although the activation of NRF2 may be regulated by multiple mechanisms, the canonical pathway involves a KEAP1-dependent activation of NRF2 [25]. Indeed, as mentioned above, before oxidative/electrophilic stress, NRF2 interacts in the cytosol with its repressor KEAP1, a thiol-rich protein containing highly reactive cysteines. However, KEAP1 is not only a factor involved in regulating NRF2 inhibition and turnover, but it may also be a sensor of oxidative stimuli. Under homeostatic conditions, KEAP1 can interact with the Cullin 3 (Cul3)-based ubiquitin ligase E3 complex that leads to the proteasomal degradation of NRF2. Conversely, increased oxidative/electrophilic stress can induce modifications in specific cysteine residues, causing a conformational change in the NRF2-KEAP1 complex that prevents the ubiquitylation of de novo NRF2 [26].

**Fig. 2:**
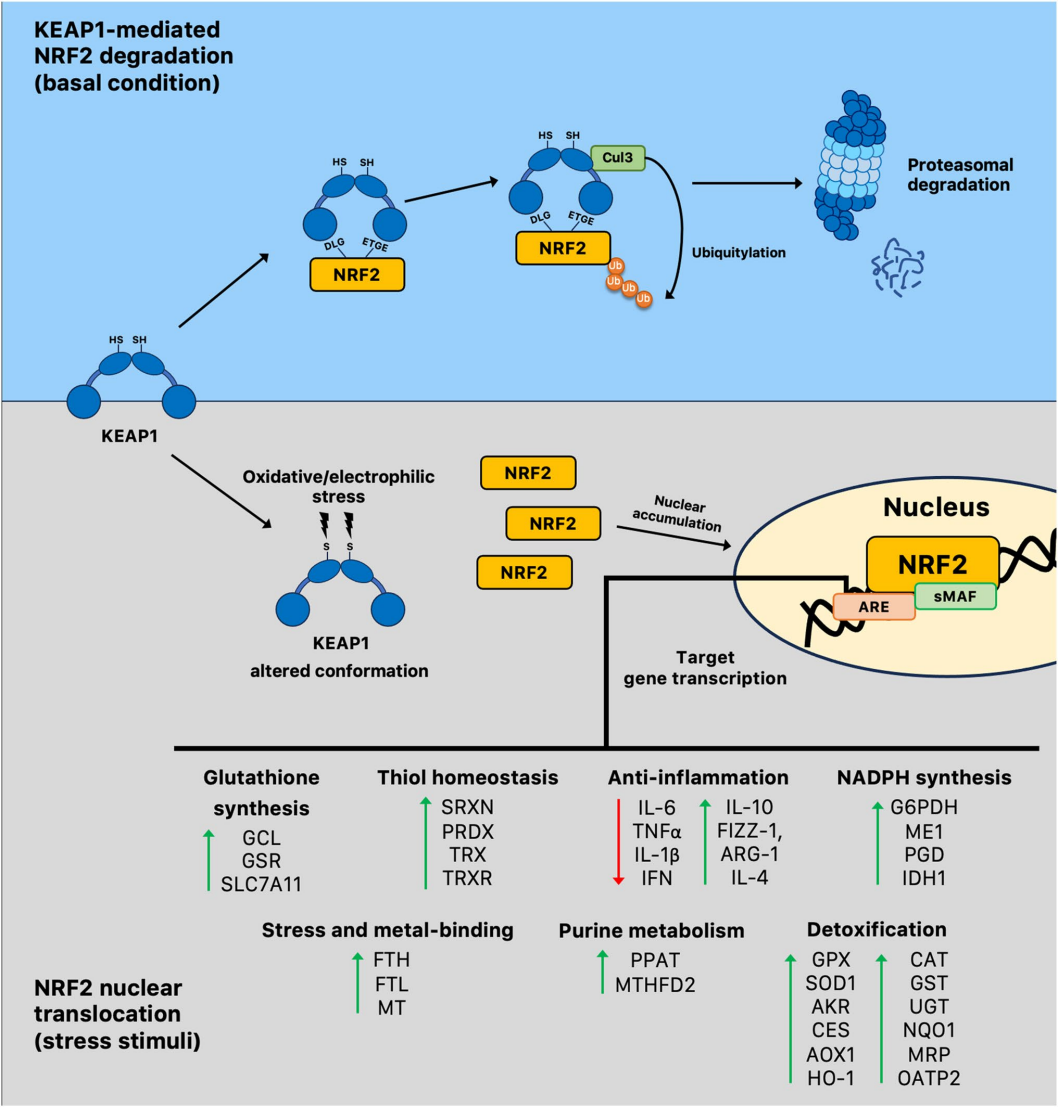
Repression and activation of NRF2 transcriptional network. NRF2 binds to KEAP1 under basal conditions, resulting in its rapid proteasomal degradation. The canonical mechanism for NRF2 activation relies on conformational changes in the KEAP1 structure induced by stress stimuli, which prevents the KEAP1–NRF2 interaction. NRF2 accumulates in the nucleus and facilitates transcription or repression of its target genes by binding to AREs. *AKR* aldo–keto reductase, *AOX1* aldehyde oxidase, *ARE* antioxidant response elements, *ARG-1* arginase 1, *CAT* catalase, *CES* carboxylesterases, *Cul3* Cullin 3, *FIZZ-1* found in inflammatory zone 1, *FTH* ferritin heavy chain, *FTL* ferritin light chain, *G6PDH* glucose-6-phosphate dehydrogenase, *GCL* γ-glutamylcysteine synthetase, *GPX* glutathione peroxidase, *GSR* glutathione reductases, *GST* glutathione *S*-transferase, *HO1* heme oxygenase-1, *IDH1* isocitrate dehydrogenase, *IFN* interferon, *IL-1β* interleukin 1β, *IL-4* interleukin 4, *IL-6* interleukin 6, *IL-10* interleukin 10, *KEAP1* kelch-like ECH-associated protein 1, *ME1* malic enzyme 1, *MRP* multidrug resistant protein, *MT* metallothionein, *MTHFD2* methylenetetrahydrofolate dehydrogenase, *NQO1* NADPH quinone oxidoreductase-1, *OATP2* organic anion transporting polypeptide 2, *PGD* phosphogluconate dehydrogenase, *PPAT* phosphoribosyl pyrophosphate amidotransferase, *PRDX* peroxiredoxin, *SLC7A11* solute carrier family 7 member 11, *sMAF* small MAF proteins, *SOD1* Cu–Zn superoxide dismutase, *SRXN* sulfiredoxin, *TNFα* tumor necrosis factor α, *TRXR* thioredoxin reductase, *TRX* thioredoxin, *UGT* UDP-glucuronosyltransferase

**Fig. 3:**
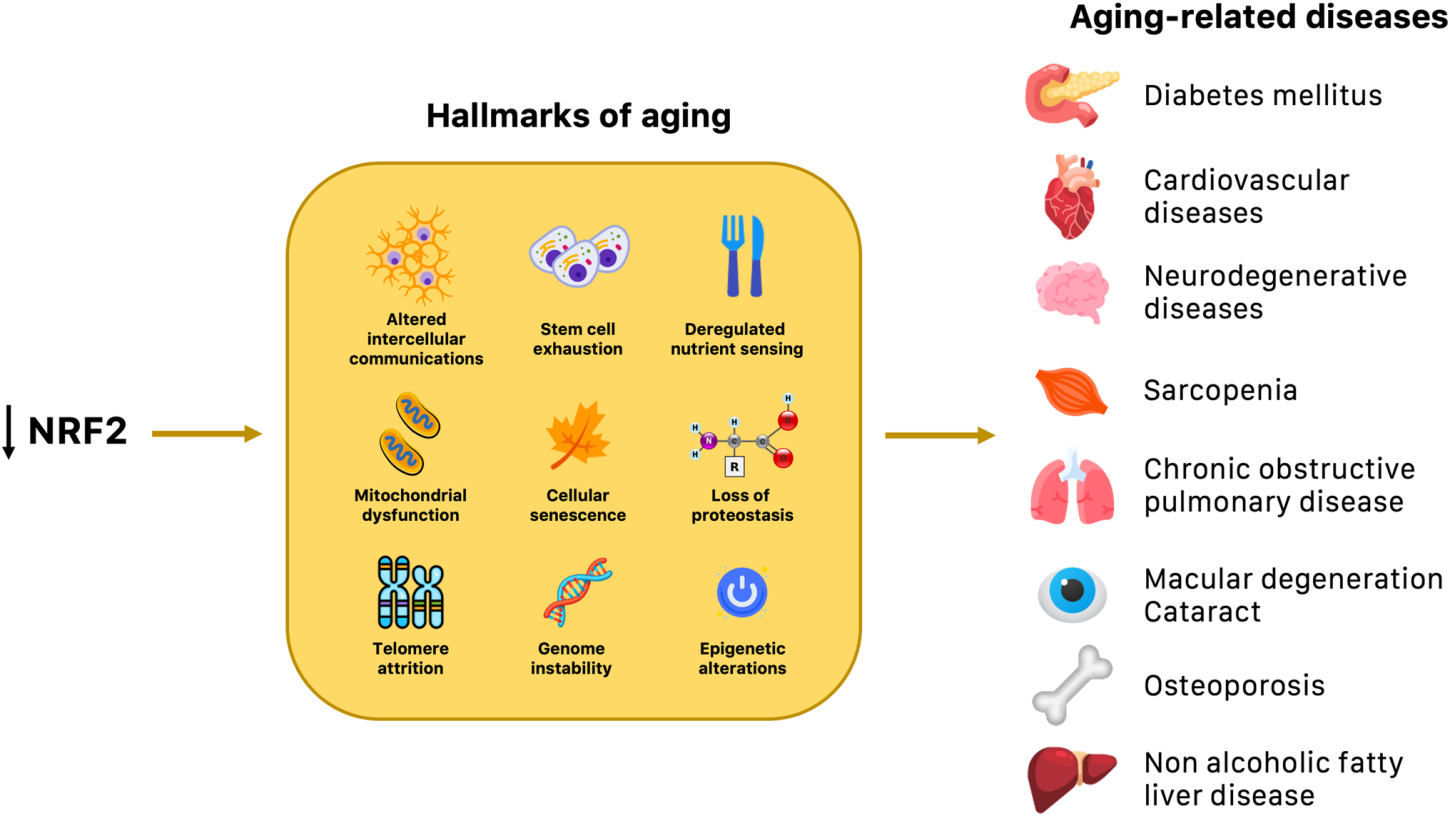
Interconnections between aging and NRF2. The age-associated decline in NRF2 activity is a critical driver of the “hallmarks of aging” contributing to the onset of diverse age-related diseases

The *cis*-acting enhancer ARE sequence is found in the promoter region of multiple genes regulated by NRF2, including various genes encoding antioxidant enzymes involved in restoring a stable internal environment after an oxidative insult. For example, NRF2 promotes the generation of nicotinamide adenine dinucleotide phosphate (NADPH), a critical cofactor that aids antioxidant reactions, by regulating the expression of genes such as glucose 6-phosphate dehydrogenase (G6PD), 6-phosphogluconate dehydrogenase (6PGD), malic enzyme 1 (ME1), and isocitrate dehydrogenase 1 (IDH1) [27]. NADPH is further used as a reducing equivalent by a large number of redox reactions, many of which are regulated by NRF2 [28]. A key mechanism involved in the restoration of redox homeostasis is the glutathione antioxidant pathway, present primarily in the reduced form [reduced glutathione (GSH)]. NRF2 controls the levels of critical enzymes involved in the synthesis of GSH, such as the catalytic and modulator subunits of glutamate–cysteine ligase (GCLC, GCLM), glutathione reductase (GR), glutathione peroxidase (GPX), and several glutathione *S*-transferases (GST) [29]. A number of studies have shown that an age-related decline in NRF2 activity can decrease the intracellular GSH pool due to reduced expression of GCLC and GCLM [30, 31]. Other important redox genes regulated by NRF2 include superoxide dismutase (SOD), catalase (CAT), heme oxygenase-1 (HO-1), NADPH quinone oxidoreductase 1 (NQO1), peroxiredoxin (PRDX), sulfiredoxin1 (SRXN1), and the thioredoxin (TRX)-based antioxidant system (Fig. 2 includes other important targets of NRF2). These enzymes participate in metabolic reactions that scavenge ROS and neutralize electrophiles, but they can undergo a gradual decline with age [32, 33]. Therefore, the impairment of NRF2 function during aging may be a key feature of a wide variety of age-related diseases (Fig. 3) characterized by elevated levels of reactive species. Moreover, accumulating data indicate that the decline of NRF2 associated with aging may exacerbate the progression of these pathologies, leading to the increased expression of pro-inflammatory biomarkers. Nuclear factor kappa B (NF-κB) is a redox-regulated transcription factor that controls the activation of many pro-inflammatory genes [34]. ROS-induced damage accumulation caused by an attenuated activity of NRF2 during aging may induce NF-κB and increase the expression pro-inflammatory mediators, such as interleukin-1-beta (IL-1β), IL-6, tumor necrosis factor-alpha (TNF-α), intercellular adhesion molecule-1 (ICAM-1), and many others [35]. It has been shown that there is a functional relationship between NRF2 and NF-κB to maintain redox homeostasis and regulate the cellular response to stress and inflammation [36]. Depletion of NRF2 may induce more aggressive inflammation through activation of NF-κB and downstream pro-inflammatory cytokines [37]. Conversely, the activation of the NRF2 pathway is often associated with a decreased activity of NF-κB resulting in attenuation of inflammatory reactions. A large number of enzymes controlled by NRF2, such as HO1, GCLC, and NQO1, may inhibit both cytokines and chemokines, including TNF-α, IL-6, IL-1β, monocyte chemoattractant protein-1 (MCP-1), and macrophage inflammatory protein-2 (MIP2) [38]. Therefore, it is clear that the crosstalk between NRF2 and NF-κB plays an important role in regulating inflammation and oxidative defense system.

## NRF2 in aging and age-related diseases

As mentioned above, the age-dependent impairment of antioxidant enzymes has been linked to a decline in the transcriptional activity of NRF2. Defective antioxidant defense mechanisms promote a pro-oxidant environment driving, at least partially, the aging phenotype and contributing to the progression of age-related diseases [39]. The imbalance between oxidants and antioxidant enzymes caused by an age-related reduction of NRF2 activity may lead to defective autophagic activities. Indeed, it has been reported that NRF2 activates both chaperone-mediated autophagy and macroautophagy, participating in the clearance of oxidized or damaged proteins during redox alterations [40, 41]. Proteotoxicity, one of the hallmarks of aging, is characterized by the accumulation of toxic misfolded proteins. An age-related decline in NRF2 function impairs the unfolded protein response (UPR), a signal transduction pathway that prevents the accumulation of misfolded proteins [42].

Aging is also characterized by a gradual decline of DNA damage repair mechanisms, resulting in an increased frequency of mutations. DNA repair proteins, such as RAD51 homolog 1 (RAD51) and p53-binding protein (53BP1), are target genes of NRF2. RAD51 and 53BP1 may facilitate the repair of DNA in both homologous recombination (HR) and non-homologous end-joining (NHEJ) [43, 44]. Therefore, reduced activity of NRF2 during aging may increase genomic instability and inhibit DNA repair mechanisms. The effect of NRF2 loss in aging could also play a role in mitochondrial dysfunction and cellular senescence. Firstly, NRF2 maintains mitochondrial function by controlling fatty acid oxidation and modulating the mitochondrial permeability transition pores (MPTP) [45]. Secondly, NRF2 can reduce the senescence-associated secretory phenotype (SASP) by modulating oxidative stress, endoplasmic reticulum stress, inflammation, immune response, and cell cycle arrest [46]. An age-related decline in NRF2 activity may also contribute to the deregulation of nutrient-sensing pathways, such as the AMP-activated protein kinase (AMPK), that are associated with aging and age-related pathogenesis [47].

Given that NRF2 controls a wide range of biological processes, including antioxidant enzyme response, a decline in its efficiency during aging may lead to the accumulation of damaged cellular components. Accordingly, unmitigated and elevated levels of ROS can increase susceptibility to neurodegeneration, cancer, and other age-related pathologies. Elevated levels of oxidation products, such as protein nitrotyrosine, carbonyls in proteins, lipid oxidation products, and oxidized DNA bases, have been observed in postmortem brains from Alzheimer’s disease (AD) patients, suggesting an increase in the oxidation of macromolecules in these patients [48]. It is also found that NRF2 nuclear localization is diminished in AD neurons and many ARE-containing genes are downregulated. This deficiency is also accompanied by an elevation of pro-inflammatory molecules, such as TNF-α, IL-1β, and IL-6 [49, 50]. Moreover, NRF2 reduces the levels of hyperphosphorylated tau, a neuronal feature of AD pathology, by enhancing its autophagic degradation [51]. The functional decline of NRF2 and its target genes with aging appears to be a major risk factor in the progression of Parkinson’s disease (PD) [52]. NRF2 may protect dopaminergic neurons by maintaining mitochondrial quality control in neuronal cells. In PD, it has been demonstrated that many antioxidant enzymes regulated by NRF2 are sequestered in Lewy bodies and, therefore, they are unable to reduce oxidative stress associated with the loss of dopaminergic neurons in the substantia nigra [53].

It is now well established that many types of human cancer are age-related diseases. Accumulating evidence suggests that NRF2 has a contradictory role in cancers; however, it plays a key role in DNA repair mechanisms by reducing genomic instability occurring in cancer cells [54]. Accordingly, the decline of NRF2 with aging may lead to tumorigenesis by increasing DNA damage and mutations. Moreover, NRF2 target genes are also critical mediators to enhance the metabolism of pro-tumorigenic xenobiotics. A transcriptional decrease of xenobiotic-metabolizing genes may be a key driver of cancer initiation in an aging population. At the same time, NRF2 and its downstream genes are overexpressed in many human cancer tissues, providing a survival and growth advantage to cancer cells. Although NRF2 activation continues to represent a crucial strategy for preventing or slowing tumorigenesis, the temporal and contextual nature of NRF2 modulation in cancer remains a complicated balancing act. [55, 56].

Sarcopenia, a progressive disorder characterized by a significant loss of muscle mass and muscle function, is a major health condition associated with aging [57]. There is also a secondary sarcopenia caused by diseases such as cancer, chronic obstructive pulmonary disease (COPD), heart failure, renal failure, and many others [58]. A relationship between NRF2 deficiency and muscle loss has been observed during aging. Many studies have demonstrated that a decrease in NRF2 activity causes muscle loss by impairing antioxidant mechanisms and muscle regeneration. In addition, NRF2 deficiency may exacerbate sarcopenia during aging by impairing skeletal muscle mitochondrial biogenesis and dynamics [59, 60]. There is also evidence that age-related deregulation of NRF2 is implicated in the pathophysiology of various conditions, including CVD, diabetes, osteoporosis, and ocular disease [22, 61].

## Impact of oxidative damage on telomere shortening

Telomeres are specialized structures formed by telomeric DNA and binding proteins. In mammals, they are highly conserved tandem repeats of the sequence TTAGGG. Telomeres are located at the end of each chromosome arm to preserve chromosomal structural integrity and maintain genomic stability. The repetitive TTAGGG sequences are bound by a group of protective proteins known as the “shelterin” complex. A detailed description of the shelterin proteins is given in Table 1. Shelterin maintains genome integrity by capping the chromosome terminus, it is implicated in the generation of T-loops, and it controls the DNA damage response (DDR) [62, 63]. Progressive telomere shortening occurs during cell division due to the inability of the DNA polymerase to synthesize in a 3′–5′ direction, leading to the incomplete replication of the lagging strand. Telomere shortening is a major driver of cellular senescence and cellular growth arrest. These phenomena exert a tumor-suppressive effect in long-lived animals, including humans. However, telomere shortening can also lead to genomic instability and promote cancer progression [64]. Telomerase, a ribonucleoprotein (RNP) complex composed of the telomerase reverse transcriptase (TERT) and telomerase RNA component (TERC), overcomes the end-replication problem of eukaryotic chromosomes. The long non-coding RNA (lncRNA) is an essential constituent of TERC containing template for telomeric DNA synthesis in association with TERT which serves as the catalytic component [65]. A second lncRNA is the telomeric repeat-containing RNA (TERRA) which is transcribed from the subtelomeric region and helps in regulating TL [66].

**Table 1:** Components of the shelterin complex

Shelterin proteins	Functions
Telomeric repeat binding factor 1 (TRF-1)	TRF-1 recognizes and binds to the 5′-TTAGGG-3′ double-stranded telomeric repeats, contributing to the formation of T-loops and regulating telomeric DNA synthesis
Telomeric repeat binding factor 2 (TRF-2)	TRF-2 protects telomeric ends and maintains telomere length homeostasis
Repressor-activator protein 1 (RAP-1)	RAP-1 is crucial for the telomere’s formation, defense, and elongation and forms a 1:1 complex with TRF-2
TRF-1 interacting nuclear protein 2 (TIN-2)	TIN-2 has the capacity to link TRF-1 with the TRF-2/RAP-1 protein complex and bring the TPP-1/POT-1 heterodimer to the ends of telomeres. This underscores the significance of TIN-2 in orchestrating the assembly of the Shelterin complex and consequently safeguarding the integrity of telomeric ends
Protection of telomeres-1 (POT-1)	POT-1, together with TPP-1, constitutes a heterodimeric binding protein that attaches to the single-stranded 5′-TTAGGG-3′ repeats. This interaction plays a pivotal role in shaping telomeric structures. Consequently, the association between POT-1 and the telomerase enzyme facilitates the extension of chromosome ends by adding new hexanucleotides
TIN-2 and POT-1 interacting protein 1 (TPP-1)	TPP-1 forms a heterodimer with POT-1, assuming a crucial role in the recruitment of telomerase to the ends of telomeres

The semiconservative nature of DNA replication implies that telomere loss may occur in a stochastic fashion [67]. Moreover, there is a large body of literature suggesting that telomere shortening is influenced by numerous factors other than the end replication problem or difficulties associated with the DNA replication machinery [68, 69]. The highly repetitive G-rich telomeric repeats are preferred sites for production of 8-oxoguanine (8-oxoG); therefore, telomeric DNA is remarkably susceptible to oxidative stress. When telomeres reach a critically short threshold, cells undergo telomere-induced (or replicative) senescence. The formation of the adduct 8-oxoguanine (8-oxoG) in the telomeric region, one of the predominant forms of free radical-induced oxidative lesions, may lead to the production of transversions (i.e., replacement of a purine with a pyrimidine or vice versa) and promote different types of cancer. Recent data revealed that persistent accumulation of 8-oxoG at telomeres promotes telomere loss and provides a mechanism by which this common oxidative lesion may drive overall genomic instability [70]. Indeed, chronic formation and persistence of 8-oxoG at telomeres not only promote telomere shortening but also chromosome end fusions and chromatin bridges. 8-oxoG can promote end-to-end fusions by causing telomere loss and crisis, which can occur due to the false recognition of “uncapped” chromosome ends as double-strand breaks (DSBs) by DDR signaling and end-joining pathways [71]. After exposure to ROS and accumulation of 8-oxoG lesions, telomeric DNA exhibits a high frequency of single-strand break formation in the DNA backbone [72]. Likewise, oxidative base damage or base loss can also block polymerases leading to telomeric double-strand breaks. 8-oxoG in duplex DNA is primarily repaired by base excision repair (BER) but the activity of BER decreases with age in multiple tissues, contributing to age-related occurrence of cancer and other age-related pathologies [73]. Several lines of evidence also indicate that the repair of oxidative lesions is less efficient in telomeres than in the rest of the genome [74, 75]. This repair deficiency in telomeres may be due to different reasons, including the overexpression of TERF-2, a component of the shelterin complex involved in the protection of chromosome ends by promoting the formation of the T-loop structure. This telomeric protein blocks the access of DNA repair enzymes to telomeric strand breaks and inhibits the phosphorylation of enzymes involved in the DDR [76, 77]. It has also been reported that oxidized telomeric DNA, particularly a telomeric region containing 8-oxoG, cannot be extended by telomerase. In addition, 8-oxoG disrupts or alters G-quadruplex structures which are inhibitory to telomerase [78, 79].

**Fig. 4:**
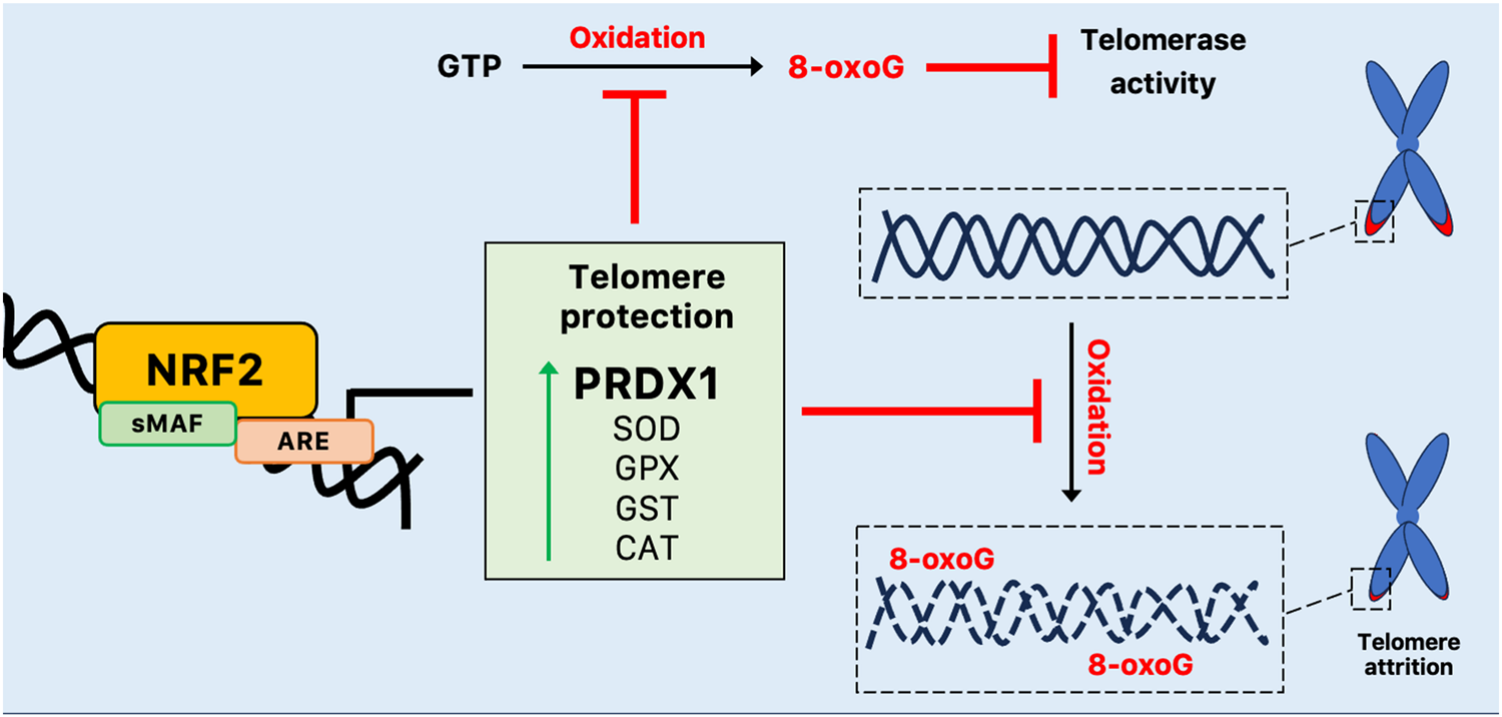
NRF2-mediated protection of telomeres from oxidative damage. NRF2-mediated transcription of PRDX1 and other cellular antioxidant defense systems diminish oxidative damage contributing to telomere protection. *8-oxoG* 8-oxoguanine, *ARE* antioxidant response elements, *CAT* catalase, *GPX* glutathione peroxidase, *GST* glutathione *S*-transferase, *GTP* guanosine-5′-triphosphate, *PRDX* peroxiredoxin, *sMAF* small MAF proteins, *SOD* Cu–Zn superoxide dismutase

Although a variety of NRF2-dependent repair proteins are involved in processing oxidative base damage, the antioxidant enzyme PRDX1 may be recruited to telomeres during the S phase of the cell cycle, reducing the abundance of ROS near telomeres and preserving telomeric DNA for extension by telomerase [72]. However, other cellular antioxidant defense systems, such as the glutathione system, CAT, and SOD, diminish ROS and therefore may contribute to telomere protection (Fig. 4) [80, 81].

Another important point is that the rate of telomere shortening may be further increased by inflammation. Chronic low-grade inflammation might enhance telomere attrition by increasing ROS-mediated DNA damage and accelerate the accumulation of senescent cells [68]. Senescent cells secrete a complex set of pro-inflammatory mediators, known as the senescence-associated secretory phenotype (SASP). SASP acts in a paracrine fashion and induces senescence in surrounding cells, thereby causing systemic chronic inflammation [82]. Numerous human studies have shown that the cumulative load of high inflammation markers, such as IL-6, TNF-α, ICAM-1, MCP-1, and C-reactive protein (CRP), is accompanied by increased risk for short TL or telomere dysfunction. The correlation between inflammatory molecules and shorter telomeres is thought to contribute to the aging process and related diseases [83–86].

## Telomere length and age-related diseases

Although telomere shortening is a well-known hallmark of aging, the clinical value of TL in age-related pathologies and mortality is a hotly debated topic. However, TL remains an informative marker to assess biological age especially when used along with other indices associated with epigenetic alterations, homeostatic dysregulation, and frailty [16]. Epidemiologic studies have repeatedly demonstrated that individuals with shorter telomeres had a mortality rate nearly twice that of those with longer telomeres [87]. In addition, an accelerated rate of telomere shortening may help to predict the risk of future age-related pathologies, such as neurodegenerative diseases, cancer, CVD, and osteoporosis [87–91].

In highly proliferative tissues (e.g., skin and hematopoietic system), continuous tissue renewal and low levels of telomerase in progenitor cell compartments may cause progressive telomere attrition over the years. Likewise, ROS-induced damage of telomere sequences in slowly proliferative tissues (e.g., brain, heart, and liver) accelerates attrition and uncapping over time [92]. Moreover, senescence-associated inflammation has been connected to shorter telomeres and is thought to drive multiple age-related diseases [19]. The relationship between telomere shortening, inflammation, and senescence has recently emerged as an important player in neurodegenerative diseases. It has been shown that TERT may play a significant role in T cell senescence and neurodegenerative diseases by regulating oxidative stress and inflammatory responses and slowing down telomere attrition. Neurons of TERT knockout mice showed shorter telomeres, increased production of oxidative species, and an increase in cellular oxidative damage in response to pathological tau [93, 94]. Despite this, TL is not only associated with AD risk but also with disease progression. A study from Tedone et al. indicates that late-onset AD patients with slow disease progression had shorter telomeres than patients with fast disease progression or healthy elderly controls [95]. It has also been shown that telomere shortening in T cells correlates with AD disease status, measured by mini mental status exam (MMSE) [96].

The role of shortened TL in cancer is highly complex and the relationship between oxidative damage and short telomeres in the development and perpetuation of cancer is subject to intensive research. Oxidative stress may contribute to the genome instability of cancerous cells and there is evidence that the telomeres from cancer cells (e.g., breast, colon, and prostate) are shorter than in healthy tissue from the same organ [97–99]. A number of studies found an association between increased incidence of cancer and accelerated telomere shortening. For example, the Normative Aging Study found that individuals who developed cancer during the follow-up experienced a telomere shortening rate that was twice as high as those without cancer [100]. The link between shortened telomeres and cancer risk has been subsequently validated in several meta-analyses. Specifically, compelling evidence has been gathered regarding the elevated risk of gastrointestinal, head, and neck cancers associated with shortened telomeres [101]. Despite the tendency of most studies to ignore the variation in TL across chromosomes and cells, it appears that the variability of TL may serve as a risk factor for cancer-related mortality. However, some studies did not observe significant connections between TL and cancer risk. Conversely, certain studies have reported higher TL in cancer patients compared to individuals without cancer [102, 103]. A Mendelian randomization study showed that genetically increased TL is associated with increased risk of several cancers. The protection of telomeres 1 (POT1) protein is an essential subunit of the shelterin telomere binding complex and appears to be most commonly involved in cancer. Recent clinical findings suggest POT1 mutations associated with long TL confer a predisposition to a range of benign and malignant solid neoplasms [104, 105].

Multiple prospective cohort studies have indicated that individuals with a shorter TL and a faster rate of telomere attrition are at a heightened risk of CVD, myocardial infarction, heart failure, and stroke [106–109]. Furthermore, Carty et al. found that leukocyte TL (LTL) has the potential to predict mortality in CVD patients [110]. It has also been shown that patients with acute coronary syndrome exhibit shortened telomeres, which were correlated with the presence of highly unstable atherosclerotic plaques. Additionally, these individuals also had elevated levels of oxidative stress and inflammatory mediators [111]. Various studies have indicated that diabetes mellitus, a common risk factor for CVD, is associated with shorter telomeres. Individuals who have both short telomeres and diabetes are characterized by a faster disease progression and an increased susceptibility to various diabetic complications, such as retinopathy, nephropathy, neuropathy, and peripheral vascular disease [112, 113]. Similar to numerous other tissues, bone cells also exhibit an age-related decrease in TL. Telomere shortening may play a role in the onset and progression of osteoporosis, which is a prevalent condition among elderly individuals. Despite some conflicting results, a large population-based study in 2150 women provided evidence that shortened LTL is associated with a decrease in bone mineral density and the presence of osteoporosis [88, 114].

## Influence of NRF2 on telomere length

An intriguing interplay between NRF2 and TL regulation is increasingly supported by emerging studies (Table 2). There is evidence that human immunodeficiency virus (HIV) infection is a model of accelerated aging associated with the premature development of age-related comorbidities, such as CVD, neurodegeneration, and metabolic syndrome. HIV is also accompanied by a disruption of the redox balance and a decrease in the protein function of NRF2, which promotes the acquisition of a premature senescence phenotype [115]. It has been found that antiretroviral therapy, a recognized risk factor for CVD, decreases TL by activating the serine/threonine kinase p90RSK and inhibiting NRF2-ARE activity. The involvement of p90RSK-NRF2 signaling in HIV accelerates the aging process by regulating four components of the senescent phenotype: (1) DNA damage caused by telomere shortening, leading to the induction of p16 and p21, (2) generation of ROS, (3) inflammation, and (4) impairment of efferocytosis induced by antiretroviral therapy [116].

**Table 2:** Summary of the studies showing an interplay between NRF2 and TL regulation

Studies	Mechanisms	Results	References
Davinelli et al.	HIV induces a disruption of the redox balance and a decrease in the protein function of NRF2	Acquisition of a premature senescence phenotype	[115]
Singh et al.	Antiretroviral therapy inhibits NRF2-ARE activity	Increased ROS-induced telomere shortening	[116]
Hu et al.	Antitelomerase therapy in cancer causes an adaptative response through NRF2	Restoration of telomere maintenance	[117]
Gong et al.	Human TERT interacts with the transcription factor YBX1 promoting the formation of a complex that binds to the NRF2 promoter region	Enhanced cell proliferation and telomere maintenance	[118]
Liu et al.	NRF2 activates the transcription of XRCC5, which, in turn, enhances the expression of human TERT	Enhanced DNA repair and telomere maintenance	[119]
Dong et al.	NRF2 promotes the expression of telomere-associated genes, including TERT and SLC7A11	Enhanced antioxidant defenses, telomere maintenance and protection against ferroptosis-induced injury	[120]
Wu et al.	Activation of NRF2 increases the expression of genes associated with telomere maintenance, including TERT	Enhanced telomerase activity, telomere lengthening, and protection against hypoxia-induced injury	[121]
Ahmad et al.	Bidirectional interplay between NRF2 and TERT	Enhanced cell survival and telomere maintenance	[122]
Aeby et al. and Lovatt et al.	NRF2 promotes the expression of PRDX1, a protective factor against oxidative stress	Prevention of oxidative damage to telomeres, preservation of telomeric DNA integrity, and facilitation of telomerase activity	[72, 123]
Kalo et al.	Mutant p53 attenuates the elements of the NRF2-dependent oxidative stress response	Increased telomere dysfunction	[125]
Lee et al. and Tian et al.	NRF2 mediates modulation of G-quadruplexes	Positive TL regulation	[128, 129]
Jurk et al. and Xiong et al.	Telomerase deficiency induces the downregulation of PGC1α and NRF2	Mitochondrial dysfunction, disrupted oxidative metabolism, and accumulation of ROS	[82, 130]

*ALT* alternative lengthening of telomeres, *ARE* antioxidant response elements, *HIV* Human immunodeficiency virus, *PGC1α* peroxisome proliferator-activated receptor-γ coactivator 1α, *PRDX1* peroxiredoxin 1, *ROS* reactive oxygen species, *SLC7A11* solute carrier family 7 member 11, *TERT* telomerase reverse transcriptase, *TL* telomere length, *XRCC5* X-ray repair cross-complementing 5, *YBX1* Y-box binding protein 1

The connection between NRF2 and telomeres has been revealed in the context of antitelomerase therapy, which holds promise for the treatment of cancer. This therapy may provoke alternative lengthening of telomeres (ALT), an alternative mechanism for telomere maintenance. It has been reported that NRF2 may be implicated in the adaptive response of cancer cells undergoing antitelomerase therapy, potentially influencing ALT activation and mitochondrial adaptive mechanisms [117]. An association between NRF2 and TERT has been demonstrated in many studies. Gong et al. reported that human TERT interacts with the transcription factor Y-box binding protein 1 (YBX1), promoting the formation of a complex that binds to the NRF2 promoter region. This interaction leads to increased NRF2 expression and contributes to cell proliferation [118]. It has also been found that NRF2 activates the transcription of X-ray repair cross-complementing 5 (XRCC5), a DNA repair gene, which subsequently enhances the expression of human TERT. This upregulation of XRCC5 and human TERT by NRF2 contributes to enhanced DNA repair mechanisms and telomere maintenance [119]. NRF2 may also attenuate ferroptosis, a form of regulated cell death, promoting the expression of telomere-associated genes, including TERT and solute carrier family 7 member 11 (SLC7A11). The increase of SLC7A11 by NRF2 contributes to telomere maintenance and enhances cellular antioxidant defenses, reducing oxidative stress and protecting against ferroptosis-induced injury [120]. Another recent study demonstrated that activation of NRF2 resulted in increased expression of genes associated with telomere maintenance, including TERT. This enhanced telomerase activity promoted telomere lengthening, protecting against hypoxia-induced cardiomyocyte injury [121]. Moreover, NRF2 may influence telomere maintenance through the regulation of TERT and the pentose phosphate pathway, contributing to cell survival [122]. Therefore, it is plausible that, in the context of the aging process, a reduction in NRF2-driven TERT expression could play a key role in driving telomere attrition [13].

An age-related decline in the transcriptional activity of NRF2 and its associated genes may contribute to the lack of cellular resilience against oxidative stress and influence telomere dynamics. PRDX1, a target gene of NRF2, plays a critical role in maintaining redox balance, and its decline is associated with age-related changes in the cornea [123]. It has been demonstrated that PRDX1 acts as a protective factor, preventing oxidative damage to telomeres and ensuring the preservation of telomeric DNA integrity. By preserving telomeric DNA, PRDX1 facilitates the extension of telomeres by telomerase, which plays a crucial role in maintaining TL [72, 123]. Although the mechanism is not fully understood, NRF2 may interact with p53 and indirectly modulate telomere dynamics. It has been well established that p53 plays a critical role in maintaining telomere stability, preventing the accumulation of abnormal cells during aging. Mounting evidence suggests that mutant p53 may repress the elements of the NRF2-dependent oxidative stress response, attenuating the expression of phase 2 detoxifying enzymes and promoting the accumulation of ROS-induced damage. Given that p53 governs telomere regulation via TRF-2, a major determinant of TL, mutant p53 may induce telomere dysfunction and associated cellular responses (i.e., senescence and/or apoptosis) through inhibition of NRF2 pathway [124–127].

G-quadruplexes are secondary DNA structures that have gained significant attention due to their involvement in various biological processes, including aging and oxidative stress.

The multiple regulatory functions of G-quadruplexes have been shown not only to influence telomeres and telomerase but also to affect gene transcription and translation as a result of the presence of these motifs in gene promoters and untranslated regions (UTR) and open reading frames (ORF) of RNA molecules. G-quadruplex structures have been identified within the regulatory regions of NRF2, regulating its translation during oxidative stress. Although the exact regulatory behavior has not yet been elucidated, preliminary studies suggest that NRF2-mediated modulation of G-quadruplexes may contribute to TL regulation [128, 129]. In recent years, it has also emerged that one of the important functions of NRF2 is to modulate mitochondrial function. For example, there is a reciprocal regulatory loop between NRF2 and peroxisome proliferator-activated receptor-γ coactivator 1α (PGC1α), which is the best-known mitochondrial biogenesis mediator. Telomerase deficiency-induced downregulation of PGC1α and NRF2 was accompanied by mitochondrial dysfunction and disrupted oxidative metabolism [82, 130].

## Targeting NRF2 to promote telomere maintenance

Targeting NRF2 activity and telomere maintenance mechanisms may represent an intriguing opportunity to treat the vast majority of age-related diseases. The most successful example of NRF2 modulators is dimethyl fumarate (DMF), currently the only NRF2 activator in clinical practice [131]. Activation of the NRF2 pathway by oral DMF administration alleviates oxidative stress and delays age-associated infertility in the mouse ovary. Likewise, after NRF2 activation through DMF, mRNA and protein levels of TERT, as well as mRNA amount of telomerase, were significantly elevated in the same animal model [132]. However, multiple compounds can theoretically restore NRF2 activity during aging and play a role in telomere maintenance, even in healthy individuals. Emerging evidence indicates that omega-3 (n-3) polyunsaturated fatty acids (PUFA), a large group of fatty acids with a broad spectrum of health benefits, may trigger a cytoprotective response via NRF2 activation [133]. Moreover, a recent meta-analysis of clinical trials suggests that n-3 PUFA, such as eicosapentaenoic acid (EPA; 20:5ω-3) and docosahexaenoic acid (DHA; 22:6ω-3), may potentially have clinical efficacy on telomere maintenance [14, 134]. Lifelong dietary DHA intervention enhanced the levels of NRF2 and profoundly attenuated telomere attrition, slowing the aging phenotype in telomerase-deficient mice [135].

The isothiocyanate sulforaphane is one of the most potent activators of NRF2. Many clinical trials have demonstrated that sulforaphane has the potential to prevent neoplastic effects through modulation of NRF2 activity, ameliorating a diversity of conditions characterized by oxidative and inflammatory stress [136, 137]. It has also been demonstrated that sulforaphane influences telomerase activity through epigenetic regulation by modulating histone acetylation levels and regulating DNA methyltransferases (DNMTs) activity in cancer lines [138, 139].

Cycloastragenol, a small molecule telomerase activator purified from the root of *Astragalus membranaceus*, has been shown to have beneficial effects on several conditions, including, insulin resistance, age-related macular degeneration, and CVD. Cycloastragenol not only enhances telomerase activity and elongates TL but also extends the healthspan and lifespan of mice without increasing the incidence of cancer [140]. Recently, it has been revealed that cycloastragenol functions at the intersection between NRF2, telomerase, and proteasome systems. The induction of telomerase activity by cycloastragenol is regulated by NRF2. However, this molecule not only increases the protein levels of human TERT but also its nuclear localization via upregulating heat shock protein 90 (Hsp90). In addition, by increasing NRF2 nuclear localization and activity, cycloastragenol upregulates cytoprotective enzymes and attenuates oxidative stress [141].

Curcumin, a lipophilic polyphenol compound derived from the rhizome of the turmeric plant, acts on multiple molecular targets that may play a protective role in various pathological conditions characterized by inflammation and increased oxidative stress [142]. For example, curcumin inhibits tumor progression through direct scavenging of ROS, induction of programmed cell death, and inhibition of NF-κB. Several studies have demonstrated the protective role of curcumin via NRF2 regulation, which in turn could maintain telomere integrity. Curcumin activates the NRF2 pathway and leads to the activation of antioxidant enzymes, including HO1, Hsp-70, TRX, and sirtuins [143–145]. As mentioned, the modulation of telomerase may be a promising approach for cancer treatment and anti-aging therapies. Based on the findings obtained from different studies, curcumin can both inhibit and maintain telomerase activity in a time- and dose-dependent manner. This effect is achieved through the regulation of human TERT translocation from the cytosol to the nucleus [146]. Quercetin and epigallocatechin gallate (EGCG) are other widely studied polyphenols that protect against oxidative damage through redox modulation of the NRF2 pathway. It has been reported that the antioxidant effect of these compounds may inhibit cardiac myocyte apoptosis by preventing telomere shortening and loss of TRF-2 expression. Similarly, low doses of other polyphenols, such as resveratrol, gallic acid, and kuromanin chloride, upregulate the mRNA levels of the human TERT in a hepatocellular carcinoma cell line, via induction of the NRF2 signaling pathway [147, 148].

## Conclusions and future perspective

In recent years, significant advancements have been achieved in understanding the role of NRF2 in both health and disease. By restoring redox homeostasis and scavenging excessive ROS levels, NRF2 has become an attractive target to prevent and/or ameliorate several diseases associated with oxidative stress. There is growing evidence indicating that a significant decrease in NRF2 transcriptional activity plays a crucial role in the aging process, contributing to the onset and progression of age-related pathologies. An age-dependent decline of NRF2 and increased oxidative stress may affect cellular and molecular processes known to drive aging, including telomere shortening. TL has emerged as a key biomarker of aging and age-related diseases, with shorter telomeres being associated with increased mortality and morbidity. However, the measurement of TL as a biomarker of disease onset, disease progression, and mortality is still far from being used in clinical practice. Analytical issues and pathophysiological aspects, such as the lack of protocol standardization and accurate measurement of TL, are the primary barriers that impede the broader clinical application of TL measurement. As discussed in this review, the interplay between the NRF2 pathway and TL is complex and multifaceted, with evidence suggesting that NRF2 activation can promote telomere maintenance and delay cellular senescence. Nevertheless, further research is needed to fully elucidate the mechanisms underlying the relationship between the NRF2 pathway and TL. A promising area of research may involve the development of novel strategies that target both pathways simultaneously, to delay the onset of age-related diseases and improve healthspan. Although this field is still in its infancy, recent studies have highlighted compounds that possess the ability to target both the NRF2 pathway and telomere maintenance mechanisms. Future endeavors aimed at comprehending the intricate interplay between NRF2 and TL, alongside the identification of novel compounds concomitantly modulating these processes, will play a pivotal role in designing intervention strategies promoting healthy aging.

## Data Availability

Data sharing is not applicable to this article as no datasets were generated or analyzed during the current study.

## Abbreviations

53BP1: P53-binding protein
6PGD: 6-Phosphogluconate dehydrogenase
8-oxoG: 8-Oxoguanine
AD: Alzheimer’s disease
AKR: Aldo–keto reductase
ALT: Alternative lengthening of telomeres
AMPK: AMP-activated protein kinase
AOX1: Aldehyde oxidase
ARE: Antioxidant response elements
ARG-1: Arginase 1
BER: Base excision repair
bZIP: Basic leucine zipper
CAT: Catalase
CES: Carboxylesterases
CNC: Cap ‘n’ collar
COPD: Chronic obstructive pulmonary disease
CRP: C-reactive protein
Cul3: Cullin 3
CVD: Cardiovascular disease
*DDR*: DNA damage response
DHA: Docosahexaenoic acid
DMF: Dimethyl fumarate
DNMTs: DNA methyltransferases
EGCG: Epigallocatechin gallate
EPA: Eicosapentaenoic acid
FIZZ-1: Found in inflammatory zone 1
FTH: Ferritin heavy chain
FTL: Ferritin light chain
G6PD: Glucose 6-phosphate dehydrogenase
GCL: γ-Glutamylcysteine synthetase
GCLC: Glutamate–cysteine ligase catalytic subunit
GCLM: Glutamate–cysteine ligase modifier subunit
GPX: Glutathione peroxidase
GR: Glutathione reductase
GSH: Reduced glutathione
GSR: Glutathione reductase
GST: Glutathione *S*-transferases
HIV: Human immunodeficiency virus
HO-1: Heme oxygenase-1
HR: Homologous recombination
Hsp90: Heat shock protein 90
ICAM-1: Intercellular adhesion molecule-1
IDH1: Isocitrate dehydrogenase
IDH1: Isocitrate dehydrogenase 1
IFN: Interferon
IL-10: Interleukin 10
IL-1β: Interleukin-1-beta
IL-4: Interleukin 4
IL-6: Interleukin 6
KEAP1: Kelch-like ECH-associated protein 1
lncRNA: Long non-coding RNA
MCP-1: Monocyte chemoattractant protein-1
ME1: Malic enzyme 1
MIP2: Macrophage inflammatory protein-2
MMSE: Mini mental status exam
MPTP: Mitochondrial permeability transition pores
MRP: Multidrug resistant protein
MT: Metallothionein
MTHFD2: Methylenetetrahydrofolate dehydrogenase
n-3: Omega-3
NADPH: Nicotinamide adenine dinucleotide phosphate
Neh: NRF2-ECH homology domain
NF-κB: Nuclear factor kappa B
NHEJ: Non-homologous end-joining
NQO1: NADPH quinone oxidoreductase 1
NRF2: Nuclear factor erythroid 2-related factor 2
OATP2: Organic anion transporting polypeptide 2
ORF: Open reading frames
PD: Parkinson’s disease
PGC1α: Peroxisome proliferator-activated receptor-γ coactivator 1α
PGD: Phosphogluconate dehydrogenase
POT-1: Protection of telomeres-1
PPAT: Phosphoribosyl pyrophosphate amidotransferase
PRDX1: Peroxiredoxin 1
PUFA: Polyunsaturated fatty acids
RAD51: RAD51 homolog 1
RAP-1: Repressor-activator protein 1
RARα: Retinoic acid receptor α
RNP: Ribonucleoprotein
ROS: Reactive oxygen species
SASP: Senescence-associated secretory phenotype
SLC7A11: Solute carrier family 7 member 11
sMAF: Small MAF proteins
SOD: Superoxide dismutase
SOD1: Cu–Zn superoxide dismutase
SRXN: Sulfiredoxin
SRXN1: Sulfiredoxin 1
TERC: Telomerase RNA component
TERRA: Telomeric repeat-containing RNA
TERT: Telomerase reverse transcriptase
TIN-2: TRF-1 interacting nuclear protein 2
TL: Telomere length
TNF-α: Tumor necrosis factor-alpha
TPP-1: TIN-2 and POT-1 interacting protein 1
TRF-1: Telomeric repeat binding factor 1
TRF-2: Telomeric repeat binding factor 2
TRF-2: Telomeric repeat binding factor 2
TRX: Thioredoxin
TRXR: Thioredoxin reductase
UGT: UDP-glucuronosyltransferase
UPR: Unfolded protein response
UTR: Untranslated regions
XRCC5: X-ray repair cross-complementing 5
YBX1: Y-box binding protein 1
β-TrCP: β-Transducin repeat-containing protein
